# *Dracaena arborea* improves sperm characteristics and antioxidant enzymes in adult male rats with experimental varicocele

**DOI:** 10.5935/1518-0557.20200098

**Published:** 2021

**Authors:** Pierre Watcho, Baudouin Yannick Petnga Tchatat, Patrick Brice Defo Deeh, Georges Romeo Bonsou Fozin, Modeste Wankeu-Nya

**Affiliations:** 1 Animal Physiology and Phytopharmacology Laboratory, University of Dschang, Box 67 Dschang, Cameroon; 2 Department of Animal Organisms Biology, University of Douala, P.O. BOX, 24157, Douala, Cameroon

**Keywords:** Varicocele, *Dracaena arborea*, antioxidant, rat

## Abstract

**Objective:**

We evaluated the preventive effects of aqueous and ethanolic extracts of *Dracaena arborea* on sperm characteristics and oxidative stress markers in adult male rats with varicocele.

**Methods:**

Thirty-six male Wistar rats were randomly distributed into 6 groups (6 animals/group) and treated for 30 days as follows: (1), normal rats receiving distilled water (10 ml/kg); (2), sham operated rats receiving distilled water (10 ml/kg); (3), varicocele rats receiving distilled water (10 ml/kg); (4), varicocele rats receiving vitamin E (150 mg/kg); (5-6), varicocele rats administered respectively with aqueous (500 mg/kg) and ethanolic (100 mg/kg) extracts of D. arborea. All rats (except normal and sham-operated groups) underwent varicocele induction. At the end of the treatment period, sexual organ weights, oxidative stress, sperm characteristics and some biochemical parameters were measured.

**Results:**

A significant decrease (*p*<0.01) in sperm density (137.81±7.76 *vs*. 175.83±4.86), sperm motility (55.43±4.49 *vs*. 77.96± 3.15) and sperm normality (44.75±2.80 *vs*. 79.25±1.84) was noticed in varicocele-untreated rats compared with controls. Varicocele also induced oxidative stress by decreasing superoxide dismutase (SOD) and catalase activities, and increasing malondialdehyde (MDA) levels. These alterations were prevented by D. arborea. For instance, the aqueous extract of D. arborea (500 mg/kg) significantly increased (*p*<0.05-0.001) testes and epididymis weights, sperm viability and sperm motility, while the ethanolic extract (100 mg/kg) increased sperm normality compared with varicocele-untreated rats. D. arborea extracts also decreased MDA levels, but elevated catalase activity.

**Conclusions:**

*Dracaena arborea* prevents the deleterious effects of varicocele and could be considered as an alternative treatment of this physiopathology.

## INTRODUCTION

Infertility is defined as an incapacity for a couple to give birth after one year of regular and unprotected sexual intercourse (Drissi *et al*., 2015). In Cameroon, approximately 25% of couples experience reproductive difficulties (Nana *et al*., 2011); in about 50% of cases in general, male responsibility is diagnosed (Fainberg & Kashanian, 2019). Various causes of male infertility such as genetic predisposition, lifestyle, medications (Fainberg & Kashanian, 2019) and varicocele (Bolat *et al.,* 2016) have been identified. Varicocele is characterized by abnormal tortuosity and dilation of the gonadal veins that drain the testis and is responsible for more than 15% of cases of male infertility (Jo *et al*., 2016). This prevalence increases significantly in men after puberty (Razi *et al*., 2011). Varicocele mainly affects the left testes (85%), and may be due to a higher probability of absence of left spermatic venous valves and to a low blood flow rate in the left renal vein compared with the inferior vena cava (Muratorio *et al*., 2013; Rehman *et al*., 2019). This pathology generates multiple consequences such as pain, hypoxia of the genital tissues, reflux of metabolites and hyperthermia, which then creates oxidative stress leading to testicular lesions, sperm injuries, hypoandrogenism and infertility (Muratorio *et al*., 2013; Bolat *et al*., 2016).

Several treatment options have been designed to attenuate pain, to stop the progression of the testicular lesions, to improve spermatogenesis and endocrine function of the testis; varicocelectomy, radiation, embolization, drugs (chorionic gonadotropic hormone) and sclerotherapy (Muratorio *et al*., 2013) are applied in patients suffering from varicocele. Indeed, these practices are associated with various side effects, including allergies to anaesthesia, hydrocele and infections, because the hormone therapy used is mostly from animal origin and often carries pathogenic germs. Moreover, a high recurrence rate and controversial effects on pregnancy (Zhu et al., 2019) very often follow surgery. Despite the beneficial effects of these treatment options, fewer studies have focused on the role of antioxidants as adjunct therapy along with surgery or hormonotherapy. The search for an effective alternative treatment from plants without side effects is on demand. Previous studies showed the efficacy of some plants in improving sperm parameters through antioxidant properties in experimental varicocele rats (Heidari *et al*., 2015).

*Dracaena arborea* is a plant used by traditional healers as an aphrodisiac to treat sexual dysfunctions. Previous studies carried out by our research group demonstrated that the aqueous (500 mg/kg) and ethanolic (100 mg/kg) extracts of *D. arborea* stimulate copulatory activity of normal and androgen-deprived (castrated) rats through dopaminergic and/or cholinergic pathway (Watcho *et al*., 2007), protect and regulate the increased rate of testicular germ cell death by apoptosis in streptozotocin-induced diabetic rats (Wankeu-Nya *et al*., 2013), and possess aphrodisiac property capable of alleviating erectile dysfunction caused by streptozotocin-induced diabetes in rats (Wankeu-Nya *et al*., 2014). *D. arborea* also delays the pro-ejaculatory effect of dopamine and oxytocin in the spine of male rats by preventing the contractions of the bulbospongiosus muscles through the blockade of dopaminergic and oxytocinergic receptors (Watcho *et al*., 2014). Moreover, we showed that *D. arborea* possesses androgenic properties, responsible for its alleviating effects on diabetes-induced reproductive complications in rats (Wankeu-Nya *et al*., 2019). A recent study also showed that the mixture of *Mondia whitei, D. arborea*, and *Bridelia ferruginea* possesses sexual stimulant effects in normal and prediabetic male Wistar rats (Watcho *et al*., 2019). Based on the above-mentioned findings, the present study was undertaken to evaluate the preventive effects of aqueous and ethanol extracts from the root barks of *D. arborea* on some fertility parameters of experimental varicocele rats.

## MATERIALS AND METHODS

### Plant collection, extract preparation and dose selection

*Dracaena arborea roots were harvested in Bagnoun, Nde Division in the West Region of Cameroon*. The plant was identified by Dr. Tatcham (Botany Department, Faculty of Science, University of Dschang, Cameroon) and authenticated at the National Herbarium under voucher number 25361/SFR/Cam. The roots were cut into small pieces, shade-dried, later transformed into powder using an electric grinder and the powder obtained was used to prepare the extracts.

The aqueous extract was obtained by macerating *D. arborea* powder (800g) in distilled water (5L) for 72h. After filtration, the filtrate was oven-dried at 45ºC and 39.68g of a brownish residue was obtained (extraction yield: 4.96%) (Wankeu-Nya *et al*., 2013; 2014).

For the ethanolic extract, 1kg of *D. arborea* powder was macerated in 5L of ethanol (95%) for 72 hours. After filtration, the filtrate was evaporated under reduced pressure using a rotative evaporator and 30g of the brownish residue was obtained (an extraction yield of 3%) (Wankeu-Nya *et al*., 2013; 2014).

The doses of plant extracts and vitamin E were chosen from previous studies (Watcho *et al*., 2007; Wankeu-Nya *et al*., 2013; 2014; Khosravanian *et al*., 2015). The working solutions of aqueous and ethanolic extracts were prepared in distilled water and administered at 500 mg/kg and 100 mg/kg, respectively, according to our pilot studies (Watcho *et al*., 2007; Wankeu-Nya *et al*., 2013; 2014).

### Animals

We used adult male Wistar rats (2.5 months old; 190-210g body weight) in this current study. They were obtained from the animal house of the Faculty of Science, University of Dschang, Cameroon. The animals were maintained in a standard environment (22-25ºC; approximately 12hrs of light and 12hrs of dark cycle), had food, and water ad libitum. The project was presented and validated by the Scientific Committee of the Department of Animal Biology, University of Dschang, which follows the internationally accepted standard ethical guidelines for laboratory animal use and care as described in the European Economic Community guidelines, EEC Directive 2010/63/EU, of the 22 September 2010 (European Union, 2010).

### Varicocele induction

Varicocele was induced as described by Turner (2001). Briefly, the upper left abdominal quadrant was exposed using a midline laparotomy incision, and the left renal vein was carefully dissected at the middle of the insertion of the spermatic vein. A 20-gauge needle was placed over the renal vein and tied using a silk suture. Thereafter, the needle was slowly removed and the midline incision was sutured using a silk thread. The dilation of the spermatic vein at the time of slaughtering was the criterion, which confirmed the varicocele in rats. Rats in the sham group underwent a similar procedure without renal vein ligation.

### Experimental protocol

Thirty-six animals (12 without varicocele and 24 with varicocele) were randomly distributed into 6 groups of 6 animals in each, and treated as follows: Group 1, normal rats receiving distilled water (10 ml/kg. bw); Group 2, sham operated rats receiving distilled water (10ml/kg. bw); Group 3, varicocele rats receiving distilled water (10 ml/kg. bw); Group 4, varicocele rats treated with vitamin E (150 mg/kg. bw); Groups 5 and 6, varicocele rats administered respectively with aqueous (500 mg/kg) and ethanolic (100 mg/kg) extracts of *D. arborea*. The rats were orally treated with vehicle and drugs for 30 days. At the end of the treatment period, we measured the testes and epididymis weights, sperm characteristics as well as oxidative stress-related biochemical parameters.

#### Tissue preparation and sample analysis

A day after the last treatment (day 31), all rats were sacrificed under diazepam (10 mg/kg) and ketamine (50 mg/kg) anaesthesia. Testes and epididymis were removed, cleared from adhering tissue, washed in saline solution and weighed. Relative sexual organ weights were calculated using the following formula: $\mathrm{Relative}\;\mathrm{sex}\;\mathrm{organ}\;\mathrm{weight}=\left(\mathrm{absolute}\;\mathrm{sexual}\;\mathrm{organ}\;\mathrm{weight}/\mathrm{body}\;\mathrm{weight}\right)\times 100$. The left and right epididymis were used for sperm count, sperm motility and sperm morphology. The left and right testes were homogenised in Tris buffer (PH = 7.4) to make 15% (g/ml) homogenate and 100 µl of this homogenate were used for measurement of oxidative stress markers (MDA, SOD and catalase) and total protein levels.

#### Sperm density and motility

Immediately after sacrifice, the left and right epididymides cauda of each rat were minced and thoroughly mixed in 10 ml of warm (36ºC) 0.9% NaCl. 20 µl of this mixture were transferred to a Malassez haemocytometer and examined under a light microscope (OLYMPUS, X400). Motile and non-motile spermatozoa were counted in 10 fields and the percentage of motile spermatozoa determined using the following formula:

$$\mathrm{Percentage}\;\mathrm{of}\;\mathrm{motile}\;\mathrm{spermatozoa}\left(\%\right)=\left(\mathrm{number}\;\mathrm{of}\;\mathrm{motile}\;\mathrm{spermatozoa}/\mathrm{total}\;\mathrm{number}\;\mathrm{of}\;\mathrm{counted}\;\mathrm{spermatozoa}\right)\times 100$$

For sperm density, a twenty-fold dilution was made by mixing the sperm suspension with 0.9% NaCl solution and the mixture was shacked gently. 20 µl of this mixture were transferred to a Malassez haemocytometer, observed under a light microscope (OLYMPUS, X400), and spermatozoa were counted in 10 fields (Ngoula *et al*., 2007).

#### Sperm viability and morphology

To determine sperm viability, 10 µl of sperm suspension were thoroughly mixed with 10 µl of eosin (1℅) and 30 µl of nigrosin (5%) on a slide. The eosin-nigrosin staining procedure was done in respect to the guidelines of the World Health Organization (WHO) on the examination and processing of semen published in 2010 (WHO, 2010). The mixture of stained sperm was smeared on the slide and examined under a light microscope (OLYMPUS, 40X). Ten (10) fields on the slide were selected in order to appreciate sperms that were stained pink or red (considered dead), and the unstained sperms (considered viable). The percentage of sperm vitality was expressed using the formula below:

$$\%\mathrm{SPZv}=\left(\mathrm{SPZv}/\mathrm{SPZt}\right)\;x\;100$$

Where %SPZv: percentage of viable spermatozoa; SPZn: number of viable spermatozoa; SPZt: total number of counted spermatozoa.

The sperm morphology was determined using eosin/nigrosin staining. Ten microliters of eosin (1%) and thirty microliters of nigrosin (5%) were added to 10 µl of sperm suspension. The prepared smear was used after incubation for 5 min in an oven (45ºC). Ten (10) fields on the slide were selected in order to appreciate various abnormalities of spermatozoa (head and tail abnormalities, cytoplasmic droplets, tailless spermatozoa). For each field, the percentage of normal spermatozoa was calculated and the mean percentage of all slides was determined using the following formula (Ngoula *et al*., 2007):

$$\%\mathrm{SPZn}=\left(\mathrm{SPZn}/\mathrm{SPZt}\right)\;x\;100$$

Where %SPZn: percentage of normal spermatozoa; SPZn: number of normal spermatozoa; SPZt: total number of counted spermatozoa.

#### Oxidative stress parameters

The testis was crushed in a mortar containing tampon tris, then cold centrifuged for 10 minutes at 3,000xg and 10% supernatant was collected from the homogenate. The supernatant was used for protein, MDA, SOD and catalase analysis. The proteins were measured using a commercial kit (Roche diagnostics cobas c-1111) and protocols were performed according to the manufacturer’s instructions. The MDA content was measured using thiobarbituric acid reaction (Soni *et al*., 2018). The tissue SOD and catalase activities were evaluated as described by Théophile *et al.* (2006).

#### Statistical analysis

The results are presented as mean ± S.E.M. One-way analysis of variance (ANOVA) followed by Tukey-HSD post-hoc test were used to determine statistical differences. All analysis were performed using the Statistica software (version 8.0, StatSoft, Inc., Tulsa, USA).

## RESULTS

### Effectivity of varicocele after surgery

**Figure 1:**
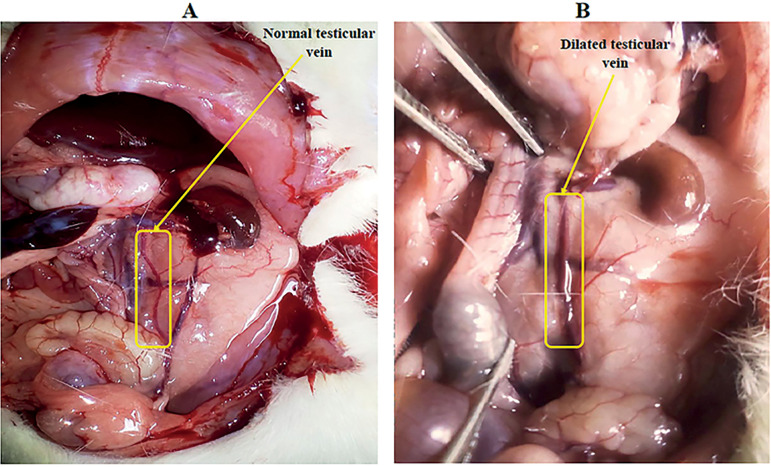
Aspect of the internal spermatic vein (A) before and (B) after partial ligation of the left renal vein.

Figure 1 shows the normal aspect of the internal spermatic vein (1A) and a dilated testicular vein (1B). Out of 32 rats that underwent varicocele induction, 24 showed a clear dilation of the left internal spermatic vein, whereas 04 cases of death and 04 cases of failure of induction were noticed, giving a success percentage of induction of 75%.

### Effect of different treatments on the relative weight of testes and epididymis

**Figure 2:**
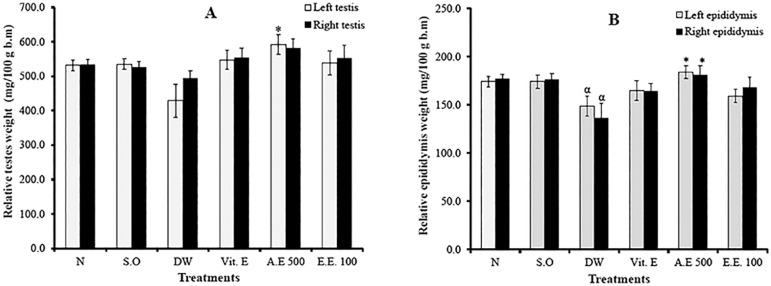
Effects of different treatments on the relative weights of testes (A) and epididymis (B). Values are mean±SEM. *:*p*<0.05 compared to distilled water; α:*p*<0.05 compared to normal; N: normal; S.O: sham operated; DW: distilled water; Vit E: vitamin E; A.E: aqueous extract; E.E: ethanolic extract.

As shown in figure 2 A-B, no statistical changes in both testicular and epididymal weights in the normal and sham operated groups were found. However, the epididymal weight (left and right) of varicocele rats receiving distilled water was significantly lowered (*p*<0.05) compared to controls. The aqueous extract of *D. arborea* significantly increased (*p*<0.05) the testicular (left testis) and epididymal (left and right epididymides) weights, compared to varicocele rats administered with distilled water (Figure 2 A-B).

### Effect of different treatments on sperm parameters

In varicocele rats treated with distilled water, sperm density (in both epididymides), sperm viability (in the left epididymis), sperm motility (in the right epididymis) and sperm normality (in both epididymides) were significantly reduced, compared with normal rats. Vitamin E significantly increased (*p*<0.05-0.001) sperm density (in both epididymis) and sperm normality (in the right epididymis), compared with varicocele rats treated with distilled water. *D. arborea* extracts improved sperm parameters after treatment. Thus, the aqueous and ethanolic extracts of *D. arborea* significantly increased sperm viability and sperm normality compared to distilled water group (Table 1).

**Table 1:** Effects of different treatments on sperm parameters

Treatments	Sperm parameters				
	Shredded epididymis	Density (*10^6^ spzs/ml)	Viability (%)	Motility (%)	Normality (%)
**Normal**	**Left**	183.54 ± 8.04	87.260 ± 2.77	72.19 ± 1.39	83.59 ± 1.70
**Sham operated**		186.88 ± 16.11	96.25 ± 2.01	73.61 ± 3.60	88.27 ± 2.26
**Distilled water**		115.42 ± 4.45^αα^, ^bbb^	87.33 ±0.88^b^	62.56 ± 4.77	64.88 ± 3.16^αα^
**Vitamin E**		158.54 ± 6.89*	94.48 ±0.77	69.76 ± 7.81	73.33 ± 1.90
**Aqueous extract (500 mg/kg)**		140.31 ± 5.47	84.94 ±1.26^##^	77.15 ± 4.11	81.63 ± 3.01*
**Ethanolic extract (100 mg/kg)**		129.06 ± 8.24	88.24 ±2.00	73.44 ± 5.21	59.94 ± 2.25^##^
**Normal**	**Right**	175.83 ± 4.86	86.40 ±1.61	77.96 ± 3.15	79.25 ± 1.84
**Sham operated**		181.77 ± 8.17	89.83 ±1.36	75.93 ± 2.93	81.87 ± 1.69
**Distilled water**		137.81 ± 7.76^αα, bb^	93.23 ±3.56	55.43 ± 4.49^αα,bb^	44.75 ± 2.80^αα, bbb^
**Vitamin E**		170.42 ± 6.18*	85.66 ±1.24	66.07 ± 5.88	73.86 ± 1.09***
**Aqueous extract (500 mg/kg)**		159.06 ± 6.62	49.11 ±0.56***, ^###^	82.84 ± 2.99***, ^#^	42.17 ± 0.70
**Ethanolic extract (100 mg/kg)**		150.00 ± 7.83	84.94 ±1.36*	74.50 ± 1.57*	77.14 ± 1.02***, ^###^

All values are expressed as mean±SEM. Number of rats per group=6.

αα *p* <0.01 compared to normal.

* *p* <0.05;

*** *p*<0.001 compared to distilled water;

# *p*<0.05;

## *p*<0.01;

### *p*<0.001 compared to vitamin E;

b *p*<0.05;

bb *p*<0.01;

bbb *p*<0.001 compared to sham operated.

### Effect of different treatments on sperm abnormalities

In the left epididymis of varicocele rats treated with distilled water, head abnormality, tail abnormality and cytoplasmic droplets were significantly increased (*p*<0.001), compared with normal rats. In the contralateral epididymis, there were no significant changes in sperm abnormalities between untreated varicocele and normal rats. The effects of vitamin E on sperm abnormalities were more pronounced in the left epididymis, where a decrease in head (23.99%) and tail abnormalities (37.99 %) was recorded compared with untreated varicocele rats. The aqueous and ethanolic extracts of *D. arborea* significantly reduced head abnormality (*p*<0.001) in the left epididymis compared to the rats in the distilled water group. The ethanolic extract significantly reduced (*p*<0.01) the cytoplasmic droplets in the left epididymis compared to those in the distilled water group. The aqueous extract was more efficient in preventing sperm abnormalities (Table 2).

**Table 2:** Effects of different treatments on sperm abnormalities

Treatments	Sperm abnormalities				
	Shredded epididymis	Head abnormality (%)	Tail abnormality (%)	Cytoplasmic droplets (%)	Tailless spermatozoa (%)
**Normal**	**Left**	7.04 ± 1.06	8.65 ± 2.35	2.43 ± 0.60	5.06 ± 1.48
**Sham operated**		3.56 ± 1.23	6.14 ± 2.25	4.64 ± 0.63	2.94 ± 0.55
**Distilled water**		14.09 ± 0.84^ααα^	19.11 ± 1.82^ααα^	6.90 ± 0.82^ααα^	7.06 ± 1.02
**Vitamin E**		10.71 ± 0.53	11.85 ± 1.14	7.53 ± 0.57	5.20 ± 1.15
**Aqueous extract (500 mg/kg)**		5.94 ± 1.00***, ^#^	16.77 ± 1.13	5.99 ± 0.81	2.41 ± 0.80
**Ethanolic extract (100 mg/kg)**		5.70 ± 1.17***, ^#^	7.40 ± 0.98^###^	2.79 ± 0.47**, ^###^	11.33 ± 1.89^#^
**Normal**	**Right**	1.61 ± 2.26	5.72 ± 0.58	5.90 ± 0.87	4.52 ± 1.07
**Sham operated**		1.36 ± 2.53	7.00 ± 0.96	6.06 ± 1.45	5.09 ± 1.09
**Distilled water**		3.56 ± 4.27	10.73 ± 4.54	7.55 ± 1.09	6.15 ± 0.46
**Vitamin E**		1.24 ± 2.51	6.66 ± 0.70	6.47 ± 1.14	5.10 ± 1.12
**Aqueous extract (500 mg/kg)**		0.56 ± 1.54*	4.51 ± 1.18	3.62 ± 0.59	3.04 ± 0.38
**Ethanolic extract (100 mg/kg)**		1.36 ± 2.74	7.65 ± 1.75	5.89 ± 1.40	5.90 ± 0.36

All values are expressed as mean±SEM. Number of rats per group=6.

ααα *p* <0.001 compared with normal;

* *p* <0.05;

** *p* <0.01;

*** *p*<0.001 compared with distilled water;

# *p*<0.05;

### *p*<0.001 compared with vitamin E.

### Effect of different treatments on biochemical parameters

**Figure 3:**
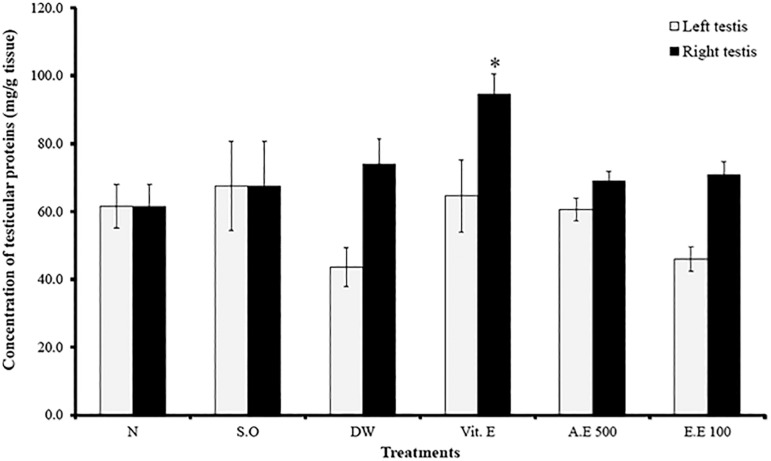
Effects of different treatments on testicular protein levels. Values are mean ± SEM. *: p<0.05 compared to distilled water; N: normal; S.O: sham operated; DW: distilled water; Vit E: vitamin E; A.E: aqueous extract; E.E: ethanolic extract.

In all varicocele rats, testicular protein levels were lowered in the left testis. Vitamin E significantly (*p* ˂0.05) increased the testicular protein content (in the right testis) after treatment (Figure 3).

**Figure 4:**
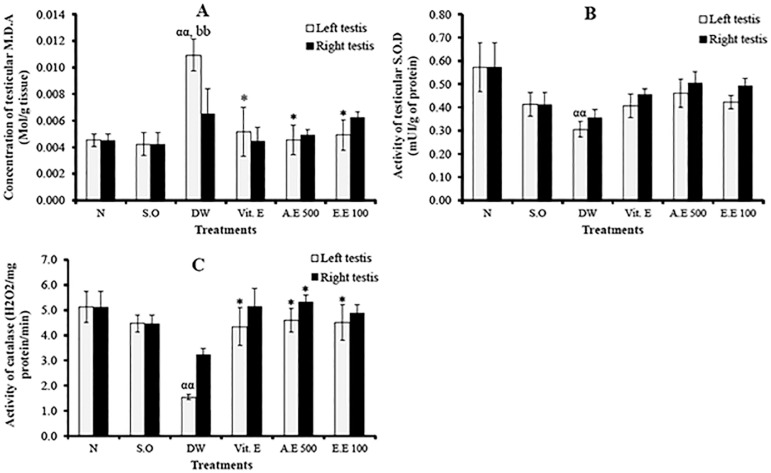
Effects of different treatments on testicular MDA (A) SOD (B) and catalase (C) activities. Values are mean ± SEM. *: *p*<0.05 compared to distilled water; ^αα^: *p*<0.01: compared to normal; ^bb^: *p*<0.01 compared to sham operated; N: normal; S.O: sham operated; DW: distilled water; Vit E: vitamin E; A.E: aqueous extract; E.E: ethanolic extract.

The induction of varicocele was associated with oxidative stress, characterized by the significant decrease (in the left testis) (*p*<0.01) of catalase and SOD activities, and elevated lipid peroxidation. In the left testis, the aqueous and ethanolic extracts of *D. arborea* significantly increased (*p*<0.05) catalase activity, but decreased MDA levels, compared with untreated varicocele animals (Figure 4).

## DISCUSSION

Varicocele is a pathology characterized by the dilation of the pampiniform plexus veins, which negatively affects testicular function (spermatogenesis and steroidogenesis), leading to infertility (Bolat *et al*., 2016). The present study clearly demonstrated that the aqueous and ethanolic extracts of *D. arborea* improved fertility potential and prevented oxidative stress. This improvement (mainly in the left testis) was characterized by the significant increase in sperm viability, sperm motility and sperm normality as well as reduced sperm abnormalities and increased antioxidant enzymes.

Varicocele appears as the result of anatomical differences between the right and left spermatic veins. Indeed, the left internal spermatic vein enters the left renal vein at a right angle, while the right internal spermatic vein enters directly into the inferior vena cava at an acute angle. This increases the hydrostatic pressure in the venous pampiniform plexus, causing its dilation (Muratorio *et al*., 2013). In this study, after surgical blockade of the left renal vein, varicocele was evidenced by the visually apparent dilation of the left spermatic vein. Out of 32 rats submitted to varicocele induction, 24 showed a clear dilation of the left internal spermatic vein, and there were 04 cases of death and 04 cases of failure, giving a success percentage of 75%. Comparatively, this percentage is lower than, the 98.3% obtained by Zhou *et al*. (2015). This difference could rely on the surgical approach used in the two studies since Zhou *et al.* (2015) used the microsurgical approach. Testicular and epididymal weights are important markers of varicocele. Similar to previous studies (Saalu *et al*., 2009), testicular and epididymal weights were lowered in varicocele rats, compared to normal animals. Data from the literature indicate that varicocele is associated with an excessive production of reactive oxygen species (ROS), which induces apoptosis in the testes and causes degeneration of the germinal epithelium of seminiferous tubuli, leading to low testicular weight (Heidari *et al*., 2015; Soni *et al*., 2018). Since the epididymal activity depends on testicular function, this drop in testicular weight may partly justify the decrease seen in the epididymal weight. These decreases in testicular and epididymal weights seen in the untreated varicocele rats are similar to the findings of Saalu *et al*. (2009). Vitamin E prevented the decline in testicular and epididymal weights; this could probably be due to its powerful antioxidant potential. In rats treated with *D. arborea* extracts, the testicular and epididymal weights were significantly increased (*p*<0.05). These results indicate that *D. arborea* could protect and/or stimulate testicular functions (steroidogenesis and spermatogenesis) as suggested by Wankeu-Nya *et al.* (2013) who showed that in streptozotocin-diabetic rats, *D. arborea* extracts protect and regulate the testicular germ cells (Wankeu-Nya *et al.*, 2013). Similarly, Heidari *et al.* (2015) reported that *Pilea microphila* significantly increased the testicular and epididymal weights of varicocele rats.

Varicocele is generally associated with abnormal sperm parameters (Yu *et al*., 2011; Muratorio *et al*., 2013). In the current study, sperm density was significantly reduced in the untreated varicocele rats. This result corroborates that of Soni *et al*. (2018) and could be justified by the adverse effects of the thermal stress, which negatively affects spermatogenesis and steroidogenesis, leading to low sperm density. Varicocele rats also exhibited low sperm motility and viability as well as elevated morphological abnormalities (Will et al., 2011; Razi *et al*., 2011). Under varicocele condition, spermatozoa, which are released in the germinal epithelium, carry more residual cytoplasm and are then considered as defective spermatozoa (Razi *et al*., 2011). Vitamin E and plant extracts prevented these abnormalities. The increase in spermatic density seen after treatment with *D. arborea* corroborates the findings of Heidari *et al*. (2015), who showed that varicocele rats treated with *Pilea microphila* exhibited a significant increase in sperm density. According to Muratorio *et al*. (2013), varicocele can slightly affect the contralateral normal testicle. *D. arborea* also improved sperm motility, viability and normality, and decreased sperm abnormalities, probably due to its antioxidant properties, which has already been previously reported (Nwaehujor *et al*., 2013). Similarly, Soni *et al.* (2018) and Zhu *et al.* (2019) showed respectively that motiliperm, a mixture of extracts of three medicinal herbs, has a protective effect in varicocele-induced oxidative injury in rat testis, and *Morinda officinalis* polysaccharides attenuate varicocele-induced spermatogenic impairment.

Several studies have reported that varicocele increases ROS production and negatively affects sperm quality (Sakamoto *et al*., 2008; Muratorio *et al*., 2013; Soni *et al*., 2018). In the present study, varicocele was associated with a significant increase in MDA concentration, but with low SOD and catalase activities. Under varicocele condition, there is an increase in testicular temperature, which causes ROS overproduction and lipid peroxidation of the spermatozoa membrane, leading to the death of germ cells (Sakamoto *et al*., 2008). SOD, an antioxidant enzyme, transforms the superoxide anion into hydrogen peroxide and oxygen, thus preventing it from exerting its noxious effects (Soni *et al*., 2018). The imbalance marked by increased MDA and decreased SOD and catalase activities due to varicocele was reversed by *D. arborea* extracts, with the highest effects observed in rats administered with the aqueous extract (500 mg/kg). In parallel, Heidari *et al.* (2015) reported that *Pilea microphila* decreased oxidative stress in the testis, by significantly increasing SOD activity after treatment. Additionally, a clinical study demonstrated that *Jingling*, a substance derived from a Chinese plant, increases catalase activity and prevents oxidative stress in men with varicocele (Yan *et al*., 2004). These protective properties of *D. arborea* extracts could be attributed to the presence of some bioactive compounds, such as sterols, phenols, flavonoids and saponins previously found in this plant (Watcho *et al*., 2007), which are powerful antioxidant compounds.

## CONCLUSION

Our findings have demonstrated that *D. arborea* improved the testicular and epididymal weights as well as sperm characteristics and antioxidant enzymes in varicocele rats; hence suggesting *D. arborea* as a reliable therapeutic plant for patients suffering from varicocele and infertility.
